# Uncovering the Relationship Between Buoyancy and Academic Achievement in Language Learning: The Multiple Mediating Roles of Burnout and Engagement

**DOI:** 10.3390/bs15101304

**Published:** 2025-09-24

**Authors:** Yicheng Cai, Honggang Liu

**Affiliations:** School of Foreign Languages, Soochow University, Suzhou 215006, China; 20234204032@stu.suda.edu.cn

**Keywords:** English learning buoyancy, English learning burnout, English learning engagement, academic achievement, mediation model

## Abstract

English learning buoyancy is a proactive and adaptable capacity that allows academic growth. However, the relationship between buoyancy, burnout, engagement, and achievement in English learning remains complex and underexplored. Grounded in the control–value theory of achievement emotions and the situated expectancy–value theory, this study investigated the impact of buoyancy and academic achievement in language learning, especially the multiple mediating roles of burnout and engagement in between. The study involved 522 senior high school students in China, who learn English as a second language. Questionnaires were employed to assess their English learning buoyancy, burnout (i.e., demotivation and exhaustion), and engagement (i.e., behavioral and agentic engagement). Academic achievement was represented by their most recent English scores. The results demonstrate that English learning buoyancy predicts academic achievement through multiple indirect paths. Specifically, exhaustion and behavioral engagement each independently mediate this relationship, and a sequential mediating pathway was identified from burnout components to behavioral engagement. The study provides pedagogical implications for English teaching.

## 1. Introduction

Leveraging the rise in positive psychology in foreign language education, which emphasizes learners’ strengths rather than their deficits (Dewaele et al., 2019; MacIntyre & Gregersen, 2012), researchers have shown increasing interest in exploring how positive cognitive and emotional factors help to sustain motivational dynamics, persist long-term endeavor, and promote psychological and academic development in language learning (Dörnyei et al., 2014; Diert-Boté & Moncada-Comas, 2024; Jin et al., 2021; H. Liu et al., 2025b; Oxford, 2016; Ushioda, 2008; Xu, 2024). This line of inquiry has brought to the forefront key concepts such as academic buoyancy—the capacity to manage the ups and downs of everyday English learning, sustain effort, and effectively overcome setbacks (Martin & Marsh, 2008; Yun et al., 2018). This daily resilience showcases positive motivational traits like sustainability, persistence, self-efficacy, and self-regulation (Jahedizadeh et al., 2019; Martin & Marsh, 2008).

Prior studies in language education revealed that buoyancy contributes to academic achievement (e.g., Derakhshan & Fathi, 2025; X. Li et al., 2023; Zhou & Wang, 2024) and emotional well-being (e.g., Man & Liu, 2025). Furthermore, buoyancy has been found to be negatively associated with burnout (Fu & Qiu, 2024; H. Liu & Cai, 2025) and positively related to engagement (H. Liu et al., 2024b; X. Wang & Hui, 2024). Language learners are especially at risk of burnout, which is described as feelings of demotivation and fatigue that students experience during English learning activities, possibly hindering achievement (Karimi & Fallah, 2021; H. Liu & Zhong, 2022). English learning engagement, another potential indicator of language learning achievement, refers to the extent of a student’s active involvement in the language learning process (Mercer, 2019; Reeve, 2012).

While empirical evidence has demonstrated pairwise associations among buoyancy, burnout, and engagement in language learning (e.g., Fu & Qiu, 2024; H. Liu et al., 2024b; Tsang & Dewaele, 2023), the intricate interaction and combined impact of these factors on English achievement remains insufficiently understood. To address this gap, the present study examines how English learning buoyancy relates to academic achievement, with a particular focus on the potential multiple mediating roles of burnout and engagement.

## 2. Literature Review

### 2.1. English Learning Buoyancy

English learning buoyancy, or academic buoyancy in English learning, is defined as ‘the capacity to negotiate the ups and downs of everyday language learning, sustain prolonged effort, and overcome setbacks on the path to L2 learning success’ (Yun et al., 2018, p. 806). Developed within a positive psychology context (Martin & Marsh, 2008), buoyancy is conceptually distinct from academic resilience, a cognate construct which emphasizes overcoming severe or chronic adversities (Martin & Marsh, 2009; M. C. Wang et al., 1994). Language learning involves inevitable day-to-day challenges, which necessitate motivation for a long-term endeavor (Diert-Boté & Moncada-Comas, 2024; H. Liu, 2024; Mohammad Hosseini et al., 2024; Oxford, 2016; G. L. Liu et al., 2024). English learning buoyancy acts as a proactive and adaptive orientation that sustains motivation dynamics in language learning, enabling learners to bounce back and step forward from everyday hassles (Dörnyei et al., 2014; Ushioda, 2008; Xu, 2024; Yun et al., 2018). Aligned with the broaden–and–build theory (Fredrickson, 2004), this positive psychological construct expands learners’ momentary thought-action repertoires, fosters long-term well-being, and contributes to learning outcomes (Martin & Marsh, 2008).

Previous studies revealed that English learning buoyancy is a positive indicator of academic achievement (e.g., Derakhshan & Fathi, 2025; X. Li et al., 2023; Yun et al., 2018; Zhou & Wang, 2024). Underpinning the capacity of buoyancy are motivational qualities such as sustainability, persistence, self-efficacy, and self-regulation (Jahedizadeh et al., 2019; Martin & Marsh, 2008). Buoyant students exhibited a greater sense of control over their cognitions, emotions, and behavior patterns in the face of academic difficulties and failures, thereby better managing time and energy in learning activities (Derakhshan & Fathi, 2025; Hirvonen et al., 2020). These cognitive-motivational mechanisms not only enhance academic outcomes but also contribute to healthy mental development. Previous studies in language learning unveiled that buoyancy can boost enjoyment (e.g., X. Wang & Hui, 2024), cultivate well-being (e.g., Man & Liu, 2025), reduce anxiety (e.g., H. Liu et al., 2024b), and mitigate boredom (e.g., Derakhshan & Fathi, 2025; Zhou & Wang, 2024). A recent study explored the complex relationships between academic buoyancy, emotions, and learning outcomes, and found that academic buoyancy in Japanese language learning promotes enjoyment and reduces boredom, thereby enhancing engagement and facilitating academic achievement (S. Han & Eerdemutu, 2025). Apart from these well-examined psychological states, recent studies in general education disclosed that students with lower levels of buoyancy are more susceptible to burnout, a maladaptive achievement emotion resulting from accumulated stress (e.g., Lakkavaara et al., 2024; Salmela-Aro & Upadyaya, 2020; Vinter, 2021). These findings highlight the pivotal role of buoyancy in mobilizing psychological resources, enhancing positive emotional experiences, and mitigating negative emotions, thereby functioning as a key protective factor in sustained language learning and in supporting learners’ enduring psychological well-being.

### 2.2. English Learning Burnout

Burnout refers to “a syndrome of emotional exhaustion and cynicism that occurs frequently among individuals who do ‘people-work’ of some kind” (Maslach & Jackson, 1981, p. 99). It is regarded as a special response, or specifically, psychological withdrawal in the process of coping unsuccessfully with accumulated stress, which affects individuals’ motivation, performance, and well-being (Cherniss, 1985; Farber, 1991; Jennett et al., 2003). The concept was introduced to the field of education (Schaufeli et al., 2002) because students also experience burnout during their learning activities, including attending classes, completing assignments, and taking exams, which are considered their ‘work’ (Q. Hu & Schaufeli, 2009; Y. Wang & Guan, 2020; Wu et al., 2024a). In language education, English learning burnout is interpreted as ‘a negative chronic psychological and emotional state’ that students feel in English learning (C. Li et al., 2021, p. 10). Researchers gradually reached a consensus that language learners are particularly vulnerable to burnout, as they encounter multiple linguistic, cultural, and psychological challenges (Karimi & Fallah, 2021; Ma et al., 2018; Wu et al., 2024b).

In psychology and education research, burnout is commonly conceptualized as a three-factor construct involving exhaustion, cynicism, and reduced efficacy (Hosseinpour Kharrazi & Ghanizadeh, 2023; C. Li et al., 2021; Schaufeli et al., 2002). In the English learning context, H. Liu and Zhong (2022) investigated burnout among Chinese senior high school students and proposed a localized two-factor model, comprising exhaustion and demotivation. Exhaustion captures the sense of fatigue learners feel during English learning tasks (C. Li et al., 2021). In contrast, demotivation refers to a decline in learners’ motivation toward English learning influenced by both internal and external factors (H. Liu & Zhong, 2022). The latter dimension resonates with the essential characteristics of cynicism and reduced efficacy described in earlier models (Maslach, 1998; Schaufeli et al., 2002).

Recent studies have investigated the associations between English learning burnout and adaptive psychological constructs. For instance, burnout has been shown to be negatively related to buoyancy (Fu, 2023), resilience (Y. Wang et al., 2024), and grit (Fan et al., 2024) in English learning. Moreover, explorations were made regarding the relationship between burnout and English learning outcome, including engagement and test achievement. Y. Wang et al.’s (2024) qualitative study regarded burnout and engagement as two spectrums of emotional experiences in language learning. Similarly, Fan et al. (2024) reported a negative correlation between burnout and engagement among high school students, and that burnout mediated the effect of L2 grit on engagement. In addition, although previous studies have revealed the adverse effects of English learning burnout on academic achievement, empirical evidence concerning high school learners remains scarce (e.g., H. Liu et al., 2024a), as most research has centered on university-level students (e.g., Hosseinpour Kharrazi & Ghanizadeh, 2023; Zhang et al., 2024; Zhou & Wang, 2024).

### 2.3. English Learning Engagement

Student engagement (i.e., school/academic engagement, see Reschly & Christenson, 2022) generally refers to students’ involvement in schooling, academics, or learning (Bempechat & Shernoff, 2012; Reeve, 2012). It has long been a subject of scholarly inquiry in the field of education, with its definition varying by research focus (Christenson et al., 2012; Philp & Duchesne, 2016). In the field of language education, engagement is a burgeoning and evolving area of research (e.g., Y. Han, 2017; H. Liu et al., 2024b; Philp & Duchesne, 2016), as language learners must actively involve in meaningful language learning activities over an extended period to develop their skills and achieve their goals (C. Liu et al., 2016; Wu et al., 2024b).

Engagement was understood as a multidimensional construct in both general and language education, typically comprising behavioral, emotional, and cognitive components (Fredricks et al., 2004; Gladstone et al., 2022; Kirkpatrick et al., 2025; Mercer, 2019; Reeve, 2012). Among these, behavioral engagement refers to students’ attention, effort, persistence, and the absence of disruptive behavior in learning (Fredricks et al., 2004; Reeve, 2012). It has received particular attention in empirical studies, partly because it is more visible and easier to assess than other dimensions (Reschly & Christenson, 2022). In fact, behavioral engagement was the earliest dimension to be conceptualized and examined in engagement research, especially within the field of motivation, where it was often described as ‘energy in action’ (Russell et al., 2005, p. 4) or seen as the behavioral expression of motivation (Skinner et al., 2009). Drawing on self-determination theory, Reeve and Tseng (2011) further added agentic engagement as the fourth subdimension to reflect students’ proactive contribution to learning, which has been widely applied in the English learning context (e.g., H. Liu et al., 2024b, 2025c; Sadoughi et al., 2023; Yang & Liang, 2025).

Previous studies in language education have emphasized engagement as a key educational outcome (Reeve & Tseng, 2011; Y. Wang et al., 2024; Zhao et al., 2025) and examined its links with psychological factors and academic achievement. Recent research shows that higher levels of engagement are associated with greater buoyancy (e.g., Fu & Qiu, 2024; H. Liu et al., 2024b; Y. Wang & Liu, 2022), self-efficacy (e.g., Derakhshan & Fathi, 2023), growth language mindset (e.g., Jiang et al., 2024; Sadoughi & Hejazi, 2023), and enjoyment (e.g., Derakhshan & Noughabi, 2024; Yang & Liang, 2025), while lower engagement is related to greater burnout (e.g., Fan et al., 2024; Fu & Qiu, 2024; Y. Wang et al., 2024) and boredom (e.g., S. Han & Eerdemutu, 2025; Tsang & Dewaele, 2023; Zhao & Wang, 2024). Engagement has also been linked to English academic achievement, though evidence remains mixed. Some studies reported a positive association (e.g., Feng & Hong, 2022; Tsang & Dewaele, 2023), while Guo et al. (2022) found that only individual-based cognitive engagement predicted test scores. Despite these fruitful findings, most studies approached engagement as a whole, without distinguishing subdimensions or their specific associations with other variables (S. Han & Eerdemutu, 2025; Mercer, 2019). Moreover, evidence from general education suggests that emotional and motivational constructs, as well as academic outcomes, are linked to different types of engagement in distinct ways (Reschly & Christenson, 2022). Considering the close link between engagement and its psychological predictors and the ambiguity of its impact on academic achievement in English learning, further investigations are warranted to unveil the relationship between subtypes of engagement, the psychological contributors, and academic achievement.

### 2.4. The Conceptual Model

The conceptual model of the present study is grounded in the control–value theory of achievement emotions (CVT; Pekrun, 2006) and the situated expectancy–value theory (SEVT; Eccles & Wigfield, 2020).

The CVT offers an integrative framework addressing the antecedents and outcomes of emotions experienced in achievement settings (Pekrun, 2006). It posits that control and value appraisals are the proximal antecedents of achievement emotion arousal, and that such emotions, in turn, influence learning outcomes such as engagement and achievement (Pekrun & Perry, 2014). Specifically, control appraisals refer to the cognitive perception of one’s capacity to carry out actions successfully and attain intended results, whereas value appraisals involve the perceived significance of the activity (i.e., goal relevance) and its perceived direction (i.e., positive or negative) (Pekrun, 2000; Pekrun et al., 2007; Pekrun & Perry, 2014). In the context of English learning, students who perceive low control over their past and future learning, together with a lack of positive value for learning activities, are more likely to experience burnout. Conversely, positive individual factors such as buoyancy, conceptualized in CVT as distal antecedents, help maintain positive appraisals and reduce negative ones, thereby lowering the likelihood of burnout and enhancing favorable learning outcomes.

Insights are also drawn from SEVT, which links expectancy and value appraisals to achievement choices, behaviors, and performance (Eccles & Wigfield, 2020). According to SEVT, learners’ expectancies for success and subjective task values (i.e., intrinsic value, attainment value, utility value, and perceived cost) predict engagement and achievement (Eccles (Parsons) et al., 1983; Eccles & Wigfield, 2020). While CVT condenses the value component into broader categories, SEVT offers a more fine-grained categorization, making it particularly suitable for the present study (Berweger et al., 2022; Putwain et al., 2018). In the English learning context, buoyant students are more likely to sustain high levels of interest and motivation, maintain the perceived value of learning activities and outcomes, and thereby sustain active engagement, which in turn fosters academic achievement. In addition, SEVT also highlights that engagement involves certain costs, particularly emotional or psychological costs associated with enduring effort and potential failure (Gladstone et al., 2022). English learning burnout can be viewed as one such emotional cost that undermines engagement. This suggests that buoyant students are less likely to experience burnout, thereby sustaining engagement and ultimately attaining higher academic achievement in English learning.

**Figure 1:**
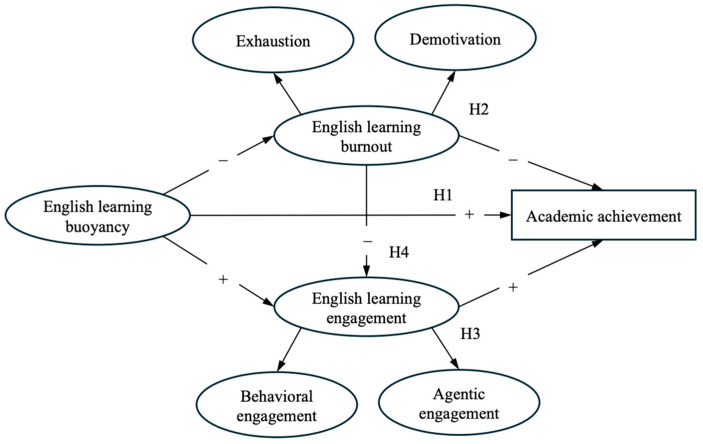
Hypothesized model of the mediation of English learning burnout and English learning engagement between English learning buoyancy and academic achievement.

Based on the above theories, the current study formulated the conceptual model (see Figure 1), with specific hypotheses outlined below.

**H1.**  *English learning buoyancy positively predicts academic achievement.*

**H2.**  *English learning burnout mediates the relationship between English learning buoyancy and academic achievement.*

**H3.**  *English learning engagement mediates the relationship between English learning buoyancy and academic achievement.*

**H4.**  *English learning burnout and engagement play a serial mediating role in the relationship between English learning buoyancy and academic achievement.*

## 3. Methodology

### 3.1. Participants

In this study, convenience sampling was used due to its ease of access to the target population (Rose et al., 2020). The current study involved 522 senior high school students from Northeast China, where English is a mandatory component of the national curriculum. All participants were second-language learners of English, having typically begun formal instruction in the third grade of primary school. The cohort included 217 males (41.6%) and 305 females (58.4%). For grade level, 204 students (39.1%) were in Grade 1, 232 (44.4%) in Grade 2, and 86 (16.5%) in Grade 3.

### 3.2. Instruments

A composite questionnaire was employed, which consists of two parts. The first section includes an introduction to the basic research information, a demographic section (e.g., gender, age, and grade), and English test scores. The second part of the questionnaire consists of three scales measuring students’ English learning buoyancy, burnout, and engagement. Participants responded to the second part on a five-point Likert scale ranging from 1 (i.e., completely disagree) to 5 (i.e., completely agree). Model fit of the measurement and structural models was evaluated according to the commonly recommended cutoff criteria proposed by L. Hu and Bentler (1999), including CFI and TLI ≥ 0.95, RMSEA ≤ 0.06, and SRMR ≤ 0.08, along with two-index combinations: CFI ≥ 0.95 with SRMR ≤ 0.09, and RMSEA ≤ 0.06 with SRMR ≤ 0.09.

#### 3.2.1. English Learning Buoyancy Scale

Participants’ buoyancy in EFL learning was measured by the four-item Academic Buoyancy Scale (Yun et al., 2018), which was developed for language learning settings. An example item is: ‘Once I decide to do something for English learning, I am like a bulldog: I don’t give up until I reach the goal’. In the current study, the scale showed satisfactory reliability (Cronbach’s α = 0.95). The measurement model exhibited satisfactory model fit, with χ^2^/df = 1.025, CFI = 1.000, TLI = 1.000, RMSEA = 0.007, and SRMR = 0.004. All standardized indicator loadings were significant (*p* < 0.001) and exceeded 0.50, suggesting good construct validity (Hair et al., 2019).

#### 3.2.2. English Learning Burnout Scale

The ten-item Senior High School English Learning Burnout Scale (H. Liu & Zhong, 2022) was employed to measure the participants’ English learning burnout. The scale has two dimensions, incorporating exhaustion (four items) and demotivation (six items). Example items include ‘the English teacher expands our extracurricular cultural knowledge related to the textbook content’ and ‘the English teacher helps me choose suitable learning materials’. In the current study, the reliability was satisfactory, with Cronbach’s α = 0.93 for exhaustion, α = 0.90 for demotivation, and α = 0.94 for the overall scale. In the confirmatory factor analysis (CFA), three items of demotivation were removed due to high modification index values (>4) and standardized residuals (>|4|) (Hair et al., 2019). Since the modifications are minor and the retained items still capture the intended dimensions, the theoretical integrity of the measurement model is largely preserved (Hair et al., 2019). After modifications, the measurement model of English learning burnout yielded satisfactory fit indices, with χ^2^/df = 2.166, CFI = 0.995, TLI = 0.992, RMSEA = 0.047, and SRMR = 0.025. All factor loadings were significant (*p* < 0.001) and above 0.50, indicating satisfactory construct validity (Hair et al., 2019).

#### 3.2.3. English Learning Engagement Scale

Participants’ engagement in English learning was assessed using the ten-item English Learning Engagement Scale (H. Liu et al., 2024b). This instrument was adapted from Reeve and Tseng’s (2011) Learner Engagement Scale by removing the emotional and cognitive dimensions and modifying item wording to align with the English learning context. The scale comprises two dimensions: behavioral engagement (five items) and agentic engagement (five items). Sample items include ‘The first time my English teacher talks about a new topic, I listen very carefully’ (behavioral engagement) and ‘During English class, I express my preferences and opinions’ (agentic engagement). The scale demonstrated excellent reliability, with Cronbach’s α = 0.94 for behavioral engagement, α = 0.94 for agentic engagement, and α = 0.94 for the global scale. After discarding two items of agentic engagement in the CFA due to large modification indices and standardized residuals (Hair et al., 2019), with χ^2^/df = 3.566, CFI = 0.989, TLI = 0.982, RMSEA = 0.070, and SRMR = 0.034. All item loadings were significant (*p* < 0.001) and higher than 0.50, providing evidence for adequate construct validity (Hair et al., 2019).

#### 3.2.4. English Academic Achievement

Academic achievement was represented by participants’ standardized scores of the most recent regional joint English examination. This comprehensive English examination covers various sections, including listening, reading comprehension, cloze test (seven choices for five blanks), gap-filling, grammar filling, and writing, with the total score being 150.

### 3.3. Data Collection and Analysis

The current study utilized the online questionnaire system Wenjuanxing (https://www.wjx.cn/ (accessed on 1 February 2025)) to distribute questionnaires to senior high school students from a city in China. The study complies with anonymity and confidentiality, and consent was obtained from both teachers and parents of the participants. Participants were encouraged to complete the questionnaire according to their real learning experiences and feelings. The online questionnaire was distributed a week after the joint examination.

Data were analyzed using IBM SPSS Statistics 27.0 and AMOS 24.0. Initial data screening excluded responses with unallowable fast completion times (faster than three seconds per item), repetitive answers (consecutive same option for over half of the items), and missing values. Mahalanobis distance was then used to remove outliers, resulting in a final set of 522 valid responses. In the primary analyses, univariate normality tests were conducted to examine the distribution of the data, with skewness and kurtosis values falling within the recommended thresholds of |3.0| and |10.0|, respectively (Kline, 2016). Item analysis was carried out separately, demonstrating satisfactory internal consistency and item discrimination of each subscale. Given that each scale was theoretically grounded, CFA with maximum likelihood estimation was performed to validate the measurement models (Yin, 2018). Convergent validity was supported by AVE values above 0.50 and composite reliability (CR) above 0.70 (Hair et al., 2019), while discriminant validity was confirmed by the Fornell–Larcker criterion, as the square root of AVE for each construct exceeded its inter-construct correlations (Fornell & Larcker, 1981). In the main analysis, descriptive statistics and correlation analyses were first conducted to provide an overview of the data. Subsequently, the hypothesized structural equation model (SEM) was tested using 5000 bootstrap resamples with bias-corrected 95% confidence intervals to obtain robust estimates of the significance of multiple mediating effects. Model fit was assessed based on the criteria described in Section 3.2 (L. Hu & Bentler, 1999).

## 4. Results

### 4.1. Descriptive and Correlation Analyses

The results of descriptive and Pearson correlation analyses among English learning buoyancy, English learning burnout (exhaustion and demotivation), English learning engagement (behavioral and agentic engagement), and academic achievement are presented in Table 1. Descriptive statistics revealed a moderate-to-high level of English learning buoyancy (M = 3.43, SD = 0.83) and global engagement (M = 3.48, SD = 0.72), and a high level of behavioral engagement (M = 3.88, SD = 0.66). Agentic engagement was at a moderate level (M = 3.09, SD = 0.96). In contrast, levels of global English learning burnout (M = 2.30, SD = 0.77) and its components (exhaustion: M = 2.14, SD = 0.84; demotivation: M = 2.46, SD = 0.86) were relatively low.

Correlations demonstrated strong connections among variables, supporting theoretical assumptions. According to Cohen’s (1988) guidelines, effect sizes (ES) of approximately 0.10, 0.30, and 0.50 are deemed as small, moderate, and large, respectively. English learning buoyancy was positively associated with global engagement and its dimensions (*r* = 0.714 to 0.816, *p* < 0.01, large ESs), and negatively associated with global burnout and its components (*r* = −0.522 to −0.457, *p* < 0.01, moderate to large ESs). Engagement was also negatively correlated with burnout (*r* = −0.419 to −0.454, *p* < 0.01, moderate ESs). Academic achievement demonstrated small but significant correlations with the psychological variables (*r* = 0.161 to 0.306, *p* < 0.01, small ESs).

### 4.2. Structural Equation Modeling

**Figure 2:**
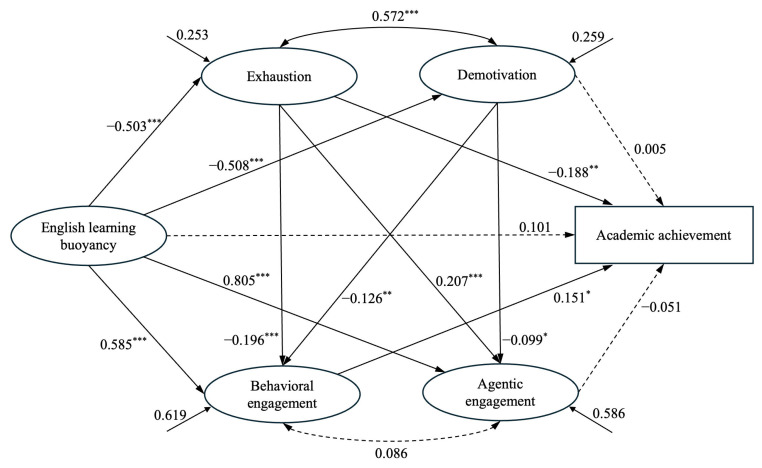
The final SEM. Note. N = 522; * *p* < 0.05, ** *p* < 0.01, *** *p* < 0.001. Dotted lines represent non-significant paths (*p* ≥ 0.05). Goodness-of-fit indices: CMIN/DF = 2.184, *p* = 0.000, CFI = 0.982, TLI = 0.978, RMSEA = 0.048, SRMR = 0.032.

The hypothesized SEM was tested, and the multiple mediating roles of burnout (exhaustion and demotivation) and engagement (behavioral and agentic engagement) were examined. The SEM (see Figure 2) exhibited a good fit to the data: CMIN/DF = 2.184, *p* = 0.000, CFI = 0.982, TLI = 0.978, RMSEA = 0.048, SRMR = 0.032, which meet the established criteria recommended by L. Hu and Bentler (1999).

The results of the path analysis are presented in Table 2. In the SEM, the direct path from English learning buoyancy to academic achievement remained statistically marginal (β = 0.101, *p* = 0.255), rejecting Hypothesis 1. However, academic achievement was negatively affected by Exhaustion (β = −0.188, *p* = 0.005 < 0.01) and positively affected by behavioral engagement (β = 0.151, *p* = 0.041 < 0.05) at a significant level. Moreover, English learning buoyancy negatively predicted exhaustion (β = −0.503, *p* < 0.001) and demotivation (β = −0.508, *p* < 0.001), while it positively impacted behavioral engagement (β = 0.585, *p* < 0.001) and agentic engagement (β = 0.805, *p* < 0.001).

Robust estimates of the indirect path from English learning buoyancy to academic achievement were provided using 5000 bootstrap resamples with the bias-corrected percentile method. The indirect effect was deemed significant if the 95% confidence interval (CI) did not include zero (Hayes, 2013). As shown in Table 2, significant mediating effects of one burnout component (exhaustion; β = 0.095, *p* = 0.013 < 0.05, CI [0.026, 0.201]) and one engagement component (behavioral engagement; β = 0.088, *p* = 0.035 < 0.05, CI [0.007, 0.219]) existed, partially supporting Hypotheses 2 and 3. Moreover, significant serial mediating effects were identified with two pathways. Buoyancy significantly predicted academic achievement through exhaustion and behavioral engagement (β = 0.015, *p* = 0.018 < 0.05, CI [0.003, 0.042]). The serial indirect effect via demotivation and behavioral engagement was significant as well (β = 0.010, *p* = 0.021 < 0.05, CI [0.001, 0.034]), partially supporting Hypothesis 4.

Robust estimates of the indirect effects from English learning buoyancy to academic achievement were obtained using 5000 bootstrap resamples with the bias-corrected percentile method. An indirect effect was considered significant if the 95% confidence interval (CI) did not include zero (Hayes, 2013). As shown in Table 2, significant mediating effects were found for one component of burnout, emotional exhaustion (*β* = 0.095, *p* = 0.013 < 0.05, CI [0.026, 0.201]), and one component of engagement, behavioral engagement (*β* = 0.088, *p* = 0.035 < 0.05, CI [0.007, 0.219]), partially supporting Hypotheses 2 and 3. In addition, two significant serial mediation pathways were identified. The serial indirect effect from buoyancy to academic achievement via exhaustion and behavioral engagement was significant (*β* = 0.015, *p* = 0.018 < 0.05, CI [0.003, 0.042]). Buoyancy also predicted academic achievement through demotivation and behavioral engagement (*β* = 0.010, *p* = 0.021, 95% CI [0.001, 0.034]). Therefore, Hypothesis 4 is partially supported.

## 5. Discussion

The current study investigated the effect of English learning buoyancy on academic achievement, particularly the multiple mediating roles of burnout and engagement. The results underscore the significance of English learning buoyancy in mitigating burnout, promoting behavioral engagement, and enhancing academic achievement. The proposed serial mediating model was supported.

While correlation analysis indicated a significant positive association between English learning buoyancy and academic achievement, SEM revealed no direct effect of buoyancy on achievement (Hypothesis 1). This finding differs from prior research that reported a direct predictive impact of buoyancy on second or foreign language achievement (e.g., Derakhshan & Fathi, 2025; Yun et al., 2018). One plausible explanation for this discrepancy lies in the inclusion of additional affective mediators, specifically learning engagement and burnout, in the present model. Most existing research has focused on the environmental contributors (e.g., Derakhshan & Fathi, 2025; H. Liu & Cai, 2025; X. Li et al., 2023) and motivational predictors (e.g., Yun et al., 2018) of buoyancy, and has emphasized the mediation of buoyancy in transmitting the effect of external support and motivation on learning achievement. However, when the focus shifted to the emotional and behavioral consequences of buoyancy in the present study, the direct impact of buoyancy on achievement waned, whereas its total effect still proved significant in the bootstrapping analysis. This finding echoes the tenets of broaden-and-build theory that positive emotions and processes potentially broaden individuals’ momentary thought–action repertoires and build enduring personal resources (Fredrickson, 2001; Martin & Marsh, 2008), underscoring that buoyancy does not act as a discrete factor that directly drives academic achievement, but operates through a complex motivational system that underpins academic success (Dörnyei, 2019; Larsen-Freeman & Cameron, 2008; Pekrun, 2024).

The mediating role of exhaustion suggests that buoyant students are less likely to suffer burnout, which in turn facilitates their academic achievement (Hypothesis 2). Echoing prior evidence that high academic buoyancy is accompanied by higher positive expectations and better emotion regulation (Hirvonen et al., 2020; Zhou & Wang, 2024), buoyant learners often frame challenges as achievable and growth-enhancing opportunities rather than as impossible hurdles (H. Liu et al., 2025a; Putwain et al., 2022; Zhao et al., 2023). As such, they are more capable of managing adverse emotions triggered by failures and difficulties in everyday English learning, preventing these negative experiences from snowballing into chronic energy depletion, which in turn sustains learning gains (Ushioda, 2008; Mohammad Hosseini et al., 2024). This finding can be interpreted through the lens of CVT (Pekrun, 2006, 2024). For senior high students in this study, English is a mandatory subject of Gaokao (National College Entrance Examination), which is widely regarded as a ‘fate-shaping’ high-stakes test and a crucial means of achieving socio-economic mobility (Fan et al., 2024; Wu et al., 2024a). Therefore, students’ extrinsic value appraisal of English achievement could be at an exceptionally high level and remains stable. However, over the extended period of English learning, students’ control appraisals, including expectancies for future achievement, attributions of past performance, and perceptions of current competence, would fluctuate in response to short-term success or failure (Dörnyei et al., 2014; Pekrun, 2024). For less buoyant students, a combination of negative control appraisal and high extrinsic value increases the risk of negative achievement emotions, such as burnout (Pekrun, 2006), a pattern also reported in Zalts et al.’s (2021) cross-cultural study among medical students. According to CVT (Pekrun, 2006), emotions are complex processes involving affective, cognitive, motivational, expressive, and physiological subsystems. Following this conceptualization, exhaustion can be regarded as the core affective component of burnout (Shirom & Melamed, 2006; Maslach & Leiter, 2016) that hinders academic achievement. In contrast, demotivation, as the other subdimension of learning burnout, primarily reflects a decline in self-efficacy and the development of cynical attitudes (Erakman & Mede, 2018; H. Liu & Zhong, 2022). This study found the mediating effect of demotivation to be marginal. While students indeed experience such negative cognitive-emotional states, these did not emerge as a significant pathway that undermines academic achievement. The high-stakes exam culture, combined with strong societal expectations, likely sustains students’ high extrinsic value and overshadows the potential detrimental effects of demotivation on achievement. By examining the mediating roles of burnout’s subcomponents, this study reveals that alleviating psychological fatigue is an effective route for channeling the benefits of buoyancy into English academic achievement among senior high school students.

The mediating role of behavioral engagement indicates that everyday resilience fosters sustained involvement in English learning, which ultimately promotes achievement (Hypothesis 3). This result echoes Putwain and Wood’s (2023) study of mathematics learning among primary school students, which substantiated that academic buoyancy indirectly predicted achievement gains via its influence on concurrent engagement. It also aligns with prior studies in language education that high levels of academic buoyancy are associated with high engagement (Fu & Qiu, 2024; Y. Wang & Liu, 2022) and that engagement contributes to achievement (Feng & Hong, 2022; Tsang & Dewaele, 2023). The mechanism of how motivational capacities translate into behavior (or engagement) and subsequently into learning or performance in the current study can be explained through the lens of expectancy–value theory (EVT; Eccles & Wang, 2012). Within the framework of EVT (or SEVT), behavioral and agentic engagement are the outcomes of students’ perceptions of task values and expectancies for success, respectively (Gladstone et al., 2022). Gladstone et al. (2022) further proposed that behavioral engagement leads to achievement-related choices and performance, while agentic engagement might not have such an effect. The results of the current study back up this proposition. Specifically, buoyant learners are more likely to maintain motivationally positive expectancies and intrinsic value appraisals of English learning activities (Yun et al., 2018; Skinner, 2019), which thereby fosters deep engagement and sustained effort, and ultimately promotes achievement in English learning (Eccles (Parsons) et al., 1983). The finding that buoyancy exerted its influence on academic achievement through behavioral rather than the agentic engagement dimension can be understood through their attribution patterns. The positivity of buoyancy indicates that buoyant students are more likely to attribute setbacks to insufficient effort, and success to effort and ability. Such a belief is reinforced by a deeply ingrained value in the Chinese educational context that hard work leads to perfection. It is also a hallmark of the growth language mindset (Dweck, 2017; H. Liu et al., 2025c; Yeager et al., 2019; Burnette et al., 2013), whose positive effects have been found to outweigh those of self-efficacy (Bai & Wang, 2020; Bai et al., 2025). Together, buoyancy is more readily translated through sustained behavioral engagement in learning tasks for enhancing achievement.

The most significant finding of the current study is the serial mediating effect of burnout (i.e., exhaustion and demotivation) and behavioral engagement in the relationship between buoyancy and academic achievement (Hypothesis 4). This sequential pathway echoes a recent study by S. Han and Eerdemutu (2025), which reported a chain mediating effect of emotions (i.e., enjoyment and boredom) and engagement between academic buoyancy and achievement in Japanese language learning among Chinese university students. The current study examined a younger cohort, affirming that buoyant students, featuring high self-efficacy, self-regulation, and proactive coping responses (Derakhshan & Fathi, 2025; Hirvonen et al., 2020; Martin & Marsh, 2008), are capable of managing adverse feelings caused by failures and difficulties in everyday English learning, demonstrating behavioral commitment to English learning, which in turn, retains enhance English learning achievement. The serial mediation mechanism through which everyday resilience mitigates burnout, fosters behavioral engagement, and facilitates achievement provides empirical support for CVT and SEVT. As mentioned in the previous paragraph, achievement emotion, such as English learning burnout, is aroused by certain combinations of control and value appraisals according to CVT (Pekrun, 2006). When facing adversities, students generate negative affective memories. However, buoyant students are capable of maintaining positive appraisals, preventing the accumulation of negative affective memories, which might induce burnout. This buffering effect of buoyancy on burnout was also reported in studies by Fu (2023) among university English learners and H. Liu and Cai (2025) among junior and senior high school students. Aligned with the proposition of CVT that achievement emotions shape both engagement and achievement, SEVT elaborates a more nuanced causal chain extending from emotion to behavioral engagement and, ultimately, to performance (Gladstone et al., 2022). Within the framework of SEVT, English learning burnout represents the psychological cost of pursuing the task, forming part of the negative value appraisal that predicts achievement behaviors. (Eccles & Wigfield, 2020; Gladstone et al., 2022). The present finding supports the postulation of SEVT, suggesting that reduced emotional costs (e.g., burnout) encourage deeper behavioral engagement. The current study contributes to the growing body of research about the complex interaction of motivational, emotional, and behavioral factors in school students’ English learning. It adds to the scant literature that highlights the mechanism of how everyday resilience benefits English learning achievement via the essential mediating role of English learning burnout and behavioral engagement.

## 6. Conclusions and Implications

This study validated the structural model of senior high school students’ buoyancy, burnout, engagement, and academic achievement in English learning. Based on the control–value theory of achievement emotions and the situated expectancy–value theory, the current study identified exhaustion and behavioral engagement as separate mediators in the relationship between English learning buoyancy and academic achievement. Furthermore, English learning burnout (i.e., exhaustion and demotivation) and behavioral engagement sequentially mediated such relationship. The findings highlight that English learning buoyancy, a positive psychological capacity marked by proactivity and adaptability, facilitates achievement through the complex interplay of cognition, motivation, emotion, and behavior.

The results yield several pedagogical implications for high school English teaching. Recognizing the importance of English learning buoyancy in mitigating negative emotions and promoting engagement and academic success, educators should focus on developing this proactive and adaptable capacity. This may involve fostering students’ positive attitudes that see adversities as results of insufficient effort rather than as a sign of fixed low ability. Students are encouraged to set small, achievable goals, such as using daily or weekly task checklists to track their progress, which boosts their sense of control, efficacy, and intrinsic motivation. Meanwhile, addressing learning burnout, particularly psychological fatigue, could be particularly effective in stressful environments with high-stakes test pressure. For instance, teachers could reduce repetitive and monotonous assignments while incorporating project-based language learning activities based on real-life scenarios that make students feel more connected to the English language. In the process, teachers could highlight their progress, which might help them regain energy through small but meaningful wins. Importantly, individual interventions are encouraged for students who are less buoyant and more prone to exhaustion, offering them sufficient support from teachers and parents. Additionally, the classroom should offer tasks of different difficulty levels to ensure all learners have opportunities for active participation, such as short comprehension checks, summarizing activities, and critical thinking exercises. In large classes, students’ engagement could be enhanced using AI-assisted tools that deliver instant feedback on quizzes or writing tasks, thereby enabling teachers to give more qualitative guidance.

The current study has several limitations. Firstly, the data in this study were derived solely from self-report measures. Future research could triangulate multiple sources of data, for instance, incorporating classroom observations to capture students’ actual behavioral responses, and interviews to gain in-depth insights into their subjective experiences. Including more diverse populations and perspectives from other stakeholders would further enhance the generalizability and richness of the findings. Secondly, the cross-sectional nature of this study precludes strong causal inference, allowing only predictive relationships. Moreover, the observed relationships may be bidirectional, as academic achievement may feedback to English learning buoyancy, burnout, and engagement according to the CVT and SEVT. Thus, longitudinal studies are recommended to examine the dynamic, reciprocal relationships among buoyancy, burnout, and engagement, and to clarify their long-term effects on English learning achievement. Thirdly, while the present study employed SEM to model linear relationships between variables, future research could adopt configurational approaches such as fuzzy-set qualitative comparative analysis to determine whether different combinations of buoyancy, burnout, and engagement may jointly result in high achievement. Finally, this study primarily focused on behavioral and agentic engagement. Future studies are encouraged to expand the investigation to include other types of engagement to gain a more comprehensive and nuanced understanding of how different engagement components interact with buoyancy and burnout in the English learning context.

## Figures and Tables

**Table 1:** Descriptive statistics and Pearson correlation coefficients.

Variable	M	SD	ELBuo	ELBur	Ex	De	ELE	BE	AE	AA
ELBuo	3.43	0.83	—							
ELBur	2.30	0.77	−0.522 **	—						
Ex	2.14	0.84	−0.490 **	0.903 **	—					
De	2.46	0.86	−0.457 **	0.909 **	0.643 **	—				
ELE	3.48	0.72	0.816 **	−0.482 **	−0.419 **	−0.454 **	—			
BE	3.88	0.66	0.714 **	−0.587 **	−0.545 **	−0.518 **	0.828 **	—		
AE	3.09	0.96	0.726 **	−0.315 **	−0.249 **	−0.321 **	0.922 **	0.547 **	—	
AA	—	—	0.255 **	−0.291 **	−0.306 **	−0.223 **	0.243 **	0.292 **	0.161 **	—

Note. ** *p* < 0.01; N = 522; ELBuo = English learning buoyancy, ELBur = English learning burnout, Ex = Exhaustion, De = Demotivation, ELE = English learning Engagement, BE = Behavioral engagement, AE = Agentic engagement, AA = Academic achievement.

**Table 2:** Direct and indirect effects of SEM analysis.

Path	β	Boot *SE*	95% CI		*p*
			Lower	Upper	
Direct effects					
Buoyancy→Ex	−0.503	0.045	−0.607	−0.426	<0.001
Buoyancy→De	−0.508	0.050	−0.689	−0.487	<0.001
Buoyancy→BE	0.585	0.040	0.396	0.554	<0.001
Buoyancy→AE	0.805	0.047	0.807	0.991	<0.001
Buoyancy→AA	0.101	0.111	−0.096	0.339	0.255
Ex→BE	−0.196	0.042	−0.238	−0.070	<0.001
Ex→AE	0.207	0.052	0.127	0.333	<0.001
Ex→AA	−0.188	0.084	−0.384	−0.049	<0.01
De→BE	−0.126	0.031	−0.149	−0.029	<0.01
De→AE	−0.099	0.051	−0.201	−0.001	<0.05
De→AA	0.005	0.064	−0.115	0.136	0.940
BE→AA	0.151	0.111	0.011	0.448	<0.05
AE→AA	−0.051	0.068	−0.187	0.080	0.462
Indirect Effects					
Buoyancy→Ex→AA	0.095	0.044	0.026	0.201	<0.05
Buoyancy→De→AA	−0.003	0.038	−0.082	0.069	0.897
Buoyancy→BE→AA	0.088	0.054	0.007	0.219	<0.05
Buoyancy→AE→AA	−0.041	0.062	−0.170	0.072	0.427
Buoyancy→Ex→BE→AA	0.015	0.010	0.003	0.042	<0.05
Buoyancy→Ex→AE→AA	0.005	0.009	−0.008	0.026	0.363
Buoyancy→De→BE→AA	0.010	0.008	0.001	0.034	<0.05
Buoyancy→De→AE→AA	−0.003	0.005	−0.018	0.003	0.270
Total Effects					
English learning buoyancy→AA	0.267	0.058	0.208	0.435	<0.001

Note. N = 522; β = Standardized path coefficient, SE = Standard Error, CI = Confidence Interval, Ex = Exhaustion, De = Demotivation, BE = Behavioral engagement, AE = Agentic engagement, AA = Academic achievement.

## Data Availability

The data presented in this study are available on request from the corresponding author.
